# Elevated expression of HSP10 protein inhibits apoptosis and associates with poor prognosis of astrocytoma

**DOI:** 10.1371/journal.pone.0185563

**Published:** 2017-10-13

**Authors:** Weibing Fan, Shuang-Shi Fan, Juan Feng, Desheng Xiao, Songqing Fan, Jiadi Luo

**Affiliations:** 1 Department of Neurology, The Third Hospital of Changsha, Changsha, Hunan, China; 2 Department of Surgery, Children′s Hospital of Hunan Province, Changsha, Hunan, China; 3 Department of Pathology, The Second Xiangya Hospital of Central South University, Changsha, Hunan, China; 4 Department of Pathology, Xiangya Hospital Central South University, Changsha, Hunan, China; Lawrence Berkeley National Laboratory, University of California, Berkeley, UNITED STATES

## Abstract

Astrocytoma is the most common type of primary malignant brain tumor, with pretty lowly 5-year survival rate in patients. Although extended surgical removal of the tumor and postoperative chemotherapy/radiotherapy executed, still there is large recurrence rate, mainly because diffuse glioma tumor cells ubiquitously infiltrate into normal parenchyma. So it becomes a priority to hunt novel molecular and signaling pathway targets to suppress astrocyma progression. HSP10, an important member of Heat shock proteins (Hsps) family, classically works as molecular chaperone folding or degradating of target proteins. Evolutionarily, HSP10 is also reported to be involved in immunomodulation and tumor progression. Poly (ADP-ribose) polymerase (PARP), important in DNA repair, is one of the main cleavage targets of caspase. And cleaved PARP (c-PARP) can serve as a marker of cells undergoing apoptosis. So far, whether the expression of HSP10 or c-PARP is associated with clinicopathologic implication for astrocytoma has not been reported. Meanwhile, it is unclear about the relationship between HSP10 and cell apoptosis. The purpose of this research is to elucidate the association between the expression of HSP10 and c-PARP and clinicopathological characteristics of astrocytoma by immunohistochemistry. The results showed that positive percentage of high HSP10 expression in astrocytoma 42/103, 40.8%) was significantly higher than that in the non-tumor control brain tissues (8/43, 18.6%) (*P* = 0.01). While no apparent difference of high c-PARP expression existed between astrocytoma and non-tumor control brain tissues. Furthermore, elevated expression of HSP10 was negative related to low expression of c-PARP (r = -0.224, *P* = 0.023), indicating high expression of HSP10 in astrocytoma inhibited apoptosis process effectively. And overexpression of HSP10 was proved to be the independent poor prognostic factor for astrocytoma by multivariate analysis. Taken together, our results suggest that elevated expression of HSP10 protein inhibits apoptosis and associates with poor prognosis of astrocytoma.

## Introduction

Astrocytoma originating in a particular kind of glial cells is the most common type of primary malignant brain tumor [1]. Then it’s reasonable that the 5-year survival rate in patients with astrocytoma is among the lowest for all cancers, literally [2]. To date, surgical removal of the tumor, chemotherapy, and radiotherapy, these conventional therapies are still the first-line clinical treatments of malignant gliomas. However, even if the great progress in understanding the biology and genetics of astrocytoma during the past decade, it hasn’t yet led to effective therapies and the prognosis of astrocytoma is still very poor [3–8]. Apparently it exaggerates the recurrence rate to a great extent that diffuse low/high-grade glioma tumor cells ubiquitously infiltrate into normal parenchyma despite extended resection. So it becomes a priority to hunt novel molecular and signaling pathway targets to suppress astrocyma progression.

HSP10, an important member of Heat shock proteins (Hsps) family, is a 10 kDa, highly conserved, mitochondrion resident protein. As we all know, HSP10, serving as “molecular chaperone”, co-chaperones with another mitochondrial heat shock protein HSP60 to assist other proteins in their folding and drive the degradation of defective proteins, which are essential for cell life and survival [9]. Evolutionarily, some “extra-chaperoning” roles of HSP10 have been gradually reported, such as participating in immunomodulation [10], cell differentiation and proliferation [11–12], especially involving in carcinogenesis [10, 13–14]. Dysplastic and neoplastic cells seem to significantly overexpress HSP10, which functionally accumulates in the cytoplasm, assisting in the transformation from dysplasia towards carcinoma, reported in several tumor and pretumoral cells [13–19].

Poly (ADP-ribose) polymerase (PARP) enzymes are a family of proteins, which partially burden vast cellular processes including gene regulation, chromatin remodeling, DNA repair and apoptosis [20]. In response to immediate cellular metabolic, chemical injury, or radiation-induced DNA SSB damage, PARP will be promptly activated by caspases- 3 and—7, resulting into cleavaged PARP(c-PARP), which is believed to be a key feature of apoptosis [21–23].

So far, whether the alteration of the expression of HSP10 or c-PARP is associated with clinicopathologic/prognostic implication for astrocytoma has not been reported. Meanwhile, it is also unclear about the relationship between HSP10 and cell apoptosis in astrocytoma patients. Here we investigated the expression of HSP10 and c-PARP proteins in the astrocytoma and in the non-tumor control brain tissues by Immunohistochemistry (IHC). We analyzed the association between expression of HSP10 and c-PARP proteins and the clinicopathological features of astrocytoma, also the correlation of HSP10 and c-PARP expression in astrocytoma.

## Materials and methods

### Ethics statement

This was a retrospective study in which we obtained these archival paraffin-embedded tissue samples during 2002–2012 from the pathology repository of Xiangya Hospital of Central South University and obtained all clinical data from patient records with approvals from the Ethics Review Board of Xiangya Hospital of Central South University (Scientific and Research Ethics Committee, No: y202/2014). Written informed consent was obtained from all patients, also the written informed consent was obtained from the next of kin, caretakers, or guardians on the behalf of the minors/children participants involved in your study.

### Tissue samples and clinical data

All these archival paraffin-embedded tumor tissue samples and non-tumor brain tissues were obtained from pathology repository, Xiangya Hospital of Central South University. These tumor samples from astrocytoma patients who were submitted to surgical treatment at the Department of Neurological Surgery at Xiangya Hospital of Central South University (Changsha, China) from 2002 to 2012. And all non-tumor brain tissues were acquired from surgical resection of normal brain tissues around astrocytomas and were also histopathologically evaluated prior to analysis. The retrospective study has been approved on March 10, 2014 by the Ethics Review Committee of Xiangya Hospital, Central South University (Scientific and Research Ethics Committee, No: y202/2014). Each patient who was enrolled in this study has signed the informed consent. These patients had a confirmed histological diagnosis of astrocytoma according to WHO histological classification of brain tumors. One hundred and three patients (54 women and 49 men, ranging in age from 8 to 70 years; mean age, 42.3) had been previously treated with chemotherapy and radiotherapy at the time of original operation. Complete clinical record and follow-up data were available for all patients. Overall survival time was calculated from the data of diagnosis to the date of death or the data last known alive. A total of 61 patients (59.2%) were alive with a mean follow-up period of 35.5 months (13–120 months).

### IHC and scores

The IHC staining for HSP10 and c-PARP proteins in glioma sections was carried out using ready-to-use Envision TM^+^ Dual Link System-HRP methods (Dako; Carpinteria, CA). As described in detail previously [24–28], the staining conditions for each antibody were adjusted according to our laboratory experience. 1:1,000 dilution of primary antibody to HSP10 protein (Mouse monoclonal antibody, Catalog: sc-376313, Santa Cruz biotechnology, Inc.) and 1:50 dilution of primary antibody to cleaved-PARP (Asp214) (Rabbit polyclonal IgG, #9541, Cell signaling, MA, USA) were applied to tissue sections to measure the target proteins expression. Positive control slides were included in every experiment in addition to the internal positive controls. The specificity of the antibody was determined with matched IgG isotype antibody as a negative control.

Immunohistochemical staining was evaluated independently by two well-trained pathologists SF and JL who were blinded to the clinicopathological data, at 400× magnification light microscopy. Positive expression of HSP10 protein was identified in cytoplasm, positive expression of c-PARP was found in the nucleus. A semi-quantitative evaluation of HSP10 was performed using a method described in the literature [29] as follows: the percentage of positive cells was divided into five grades (percentage scores): ≤10% (0), 11–25% (1), 26–50% (2), 51–75% (3), and >75% (4). The intensity of staining was divided into four grades (intensity scores): no staining (0), light brown (1), brown (2), and dark brown (3). Staining positivity was determined by the formula: overall scores = percentage score × intensity score. The sum of the staining scores was used as the final staining score for HSP10 (0–12). Expression of c-PARP protein was estimated microscopically by counting c-PARP positive cells at original magnification ×400 using the semi-quantitative method described in the literature with minor modifications [30] as follows: absent (-, no positive cells) to weak (+, 1–5 positive cells), moderate (++, 5–20 positive cells) and strong (+++, >20 positive cells) in views obtained with the areas of 10 high power fields. For statistical analysis, a final staining score of 7–12 was considered to be high expression of HSP10 protein while scores less than 7 were considered low expression of HSP10. The results of >20 c-PARP positive cells were considered to be high expression of c-PARP (high apoptotic indices, AIs) while numbers of c-PARP positive cells less than 20 were considered low expression of c-PARP (low AIs). Agreement between the two evaluators was 98%, and all scoring discrepancies were resolved through discussion between the two evaluators.

### Statistical analyses

All statistical analyses were performed using SPSS 13.0 software (SPSS, Inc.). The chi-square test was used to analyze the relationship between the expression of HSP10 and c-PARP proteins and clinicopathological characteristics and prognostic factors in NPC. The Spearman's rank correlation coefficient was used to assess the significance of the association among expression of HSP10 and c-PARP proteins in astrocytoma. Kaplan-Meier analysis was performed for overall survival curves and statistical significance was assessed using the log-rank test. Overall survival was defined as the time from the treatment initiation (diagnosis) to the date of death. To evaluate whether expression of HSP10 and c-PARP proteins are the independent prognostic factors of overall survival for astrocytoma patients, multivariate analysis using the Cox proportional hazard regression model was performed. All p-values were based on the two-sided statistical analysis and p-value less than 0.05 was considered to be statistically significant.

## Results

### Association between expression of HSP10 and c-PARP proteins and clinicopathological features of astrocytoma

**Fig 1:**
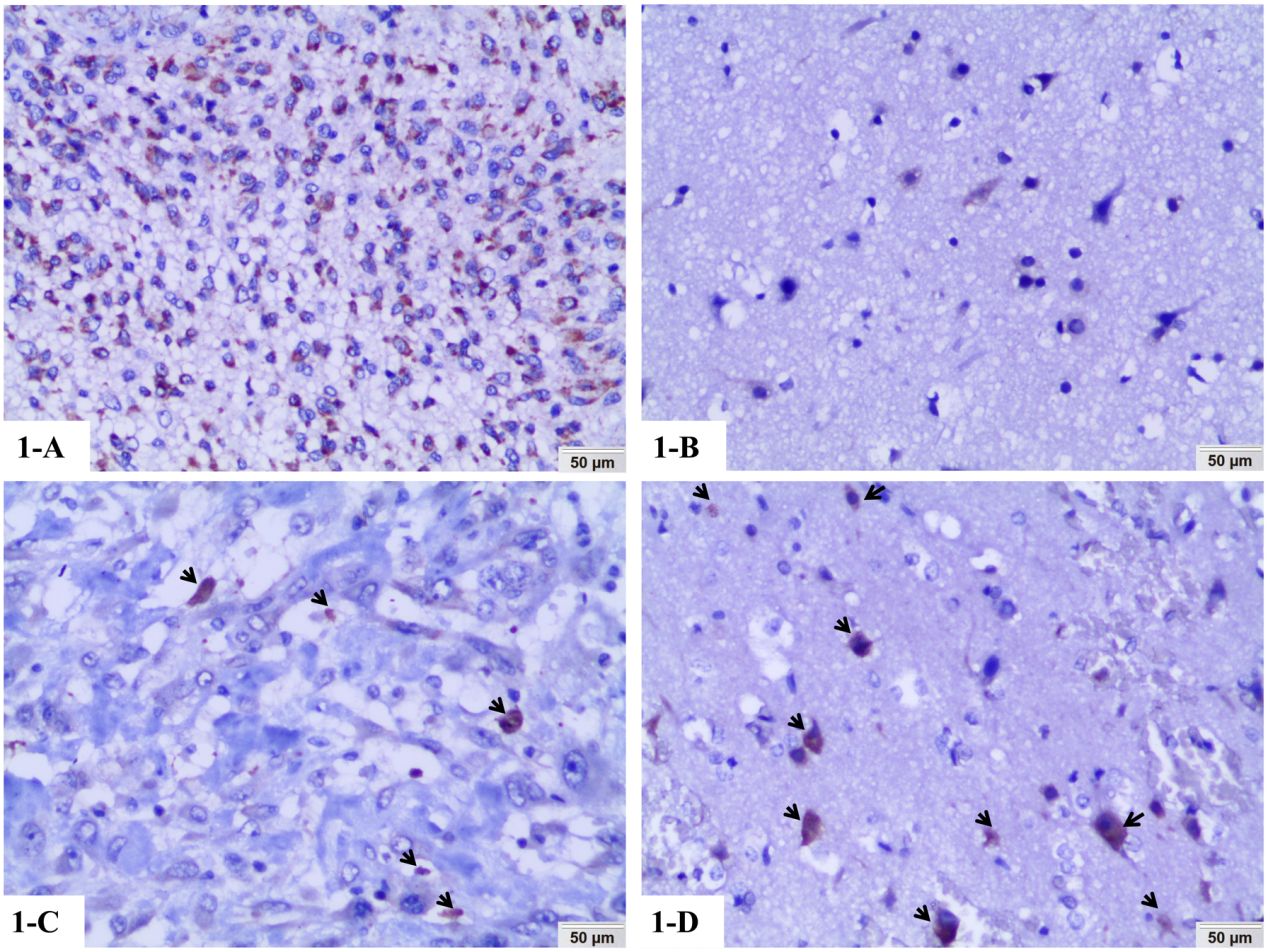
Expression of HSP10 and c-PARP proteins in astrocytoma was detected by immunohistochemistry. 1-A, B: Positive expression of HSP10 was located in the cytoplasm of astrocytoma cells (high expression) and non-tumor control brain tissues (low expression) (20x, IHC, DAB staining). 1-C, D: Positive staining of c-PARP (arrows) was identified in the nucleus of astrocytoma cells (low expression: low apoptotic indices, AIs) and in the neurons of non-tumor control brain tissues (high expression: high AIs) (20x, IHC, DAB staining).

**Fig 2:**
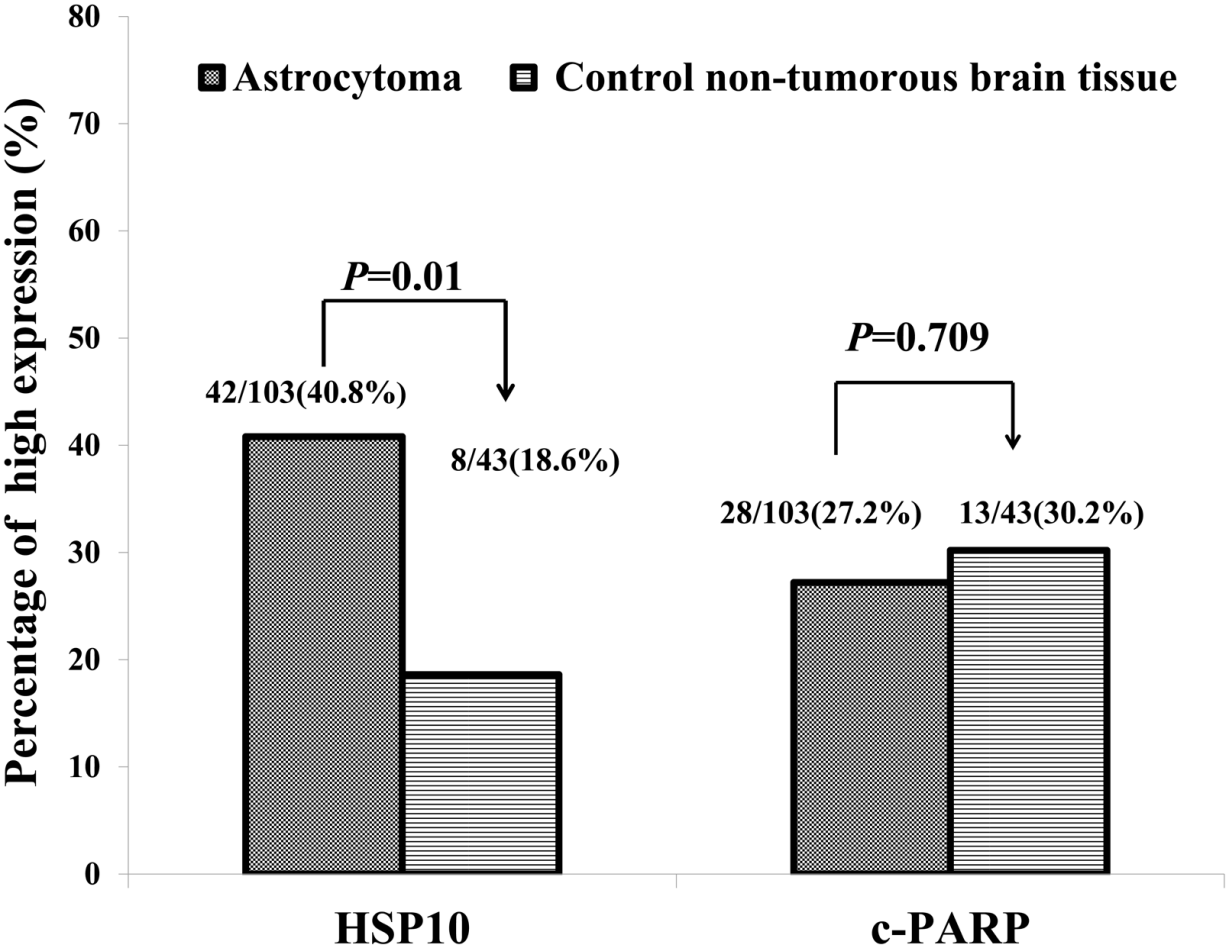
Expression of HSP10 and c-PARP proteins in astrocytoma compared to non-tumor control brain tissues. Results showed that there were significant differences between the groups which were statistically evaluated by chi-square test (*P* < 0.05).

We examined the positive expression and cellular location of HSP10 and c-PARP in astrocytoma and non-tumor control brain tissues by IHC. Positive expression of HSP10 was found in the cytoplasm of astrocytoma cells (high expression) (Fig 1A), while positive expression of HSP10 in non-tumor control brain tissues was still located in the cytoplasm but revealed very low expression (Fig 1B) (20x, IHC, DAB staining). Different to HSP10, positive expression of c-PARP was identified in the nucleus of astrocytoma cells (low expression) (Fig 1C) and non-tumor control brain tissues (high expression) (Fig 1D) (20x, IHC, DAB staining). The percentage of high expression of HSP10 and c-PARP in astrocytoma and non-tumor control brain tissues was 40.8% (42/103), 27.2% (28/103), 18.6% (8/43) and 30.2% (13/43), respectively (Fig 2). There was significantly higher percentage of high expression of HSP10 in astrocytoma compared with the non-tumor control brain tissues (*P* = 0.01). No apparent difference in the c-PARP high expression was found between astrocytoma and non-tumor control brain tissues (*P* = 0.709). We further investigated the associations between the expression of HSP10 and c-PARP proteins and clinicopathological features of astrocytoma including age, gender, tumor size, WHO grade and survival status in univariate chi-square test. These results in Table 1 promisingly displayed the tight link for clinic. A comparison of expression of the two proteins above in varying tumor sizes of astrocytoma (<5 cm or ≥5 cm) showed that significantly higher percentage of high c-PARP expression was found in larger tumor size of astrocytoma (*P* = 0.033), while HSP10 had no correlation with tumor sizes of astrocytoma. In addition, the elevated c-PARP was related to astrocytoma WHO grade (*P* = 0.029). Overexpressed HSP10, high expression of HSP10 combined with low expression of c-PARP and other immunophenotype of HSP10 and c-PARP expression had no impact on astrocytoma WHO grade. Furthermore, patients experienced recurrence, compared to replase free patients who were alive or dead, had higher high expression rates of HSP10 and high expression of HSP10/low expression of c-PARP (*P* = 0.022, *P* = 0.025 respectively). While the expression of c-PARP was no significantly associated with the recurrence. Besides, no correlation was discovered between positive expression of proteins above and age and gender (*P*> 0.05).

**Table 1:** Association between expression of HSP10 and c-PARP proteins and astrocytoma clinical pathological features (n = 103).

Characteristics (n)	HSP10			c-PARP			HSP10/c-PARP^#^		
	Low (%)	High (%)	*P-value*	Low (%)	High (%)	*P-value*	N^-^ (%)	P^+^ (%)	*P-value*
**Age**^1^									
< 43 (n = 44)	28(63.6)	16(36.4)	0.431	31(70.5)	13(29.5)	0.642	34 (77.3)	10 (22.7)	0.055
≥43 (n = 59)	33(55.9)	26(44.1)		44(74.6)	15(25.4)		35(59.3)	24 (40.7)	
**Gender**									
Female (n = 54)	33 (61.1)	21(38.9)	0.682	36 (66.7)	18 (33.3)	0.141	37 (68.5)	17 (31.5)	0.729
Male (n = 49)	28 (57.1)	21(42.9)		39(79.6)	10 (20.4)		32(65.3)	17(34.7)	
**Tumor size**^2^									
<5.0 cm (n = 58)	30 (51.7)	28 (48.3)	0.079	47 (81.0)	11 (19.0)	0.033*	35 (60.3)	23(39.7)	0.103
≥5.0 cm (n = 45)	31(68.9)	14(31.1)		28(62.2)	17 (37.8)		34(75.6)	11 (24.4)	
**WHO grade**									
I (n = 16)	11 (68.8)	5(31.2)		13 (81.3)	3 (18.7)		11 (68.8)	5 (31.2)	
II(n = 32)	21 (65.6)	11 (34.4)		28 (87.5)	4 (12.5)		19 (59.4)	13(40.6)	
_III_(n = 39)	23 (59.0)	16 (41.0)	0.234	26 (66.7)	13 (33.3)	0.029*	28 (71.8)	11(28.2)	0.731
IV (n = 16)	6 (37.5)	10 (62.5)		8(50.0)	8 (50.0)		11(68.8)	5 (31.2)	
**Survival status**									
**Replase free survival (n = 36)**	27 (75.0)	9 (25.0)		29(80.6)	7 (19.4)		30(83.3)	6 (16.7)	
Replase (n = 25)	10 (40.0)	15 (60.0)	0.022*	15(60.0)	10 (40.0)	0.203	13(52.0)	12 (48.0)	0.025*
Death (n = 42)	24 (57.1)	18 (42.9)		31(73.8)	11 (26.2)		26(61.9)	16 (38.1)	

**Abbreviations:** Statistical analysis was performed using the Chi-squared test.

*Statistically significant (p < 0.05).

^1^The average age of all subjects was 42.3 years;

^2^The average tumor size of all subjects was 5.0cm. n, Number of cases; WHO, World Health Organization.

^#^ N^-^: Other immunophenotype of HSP10 and c-PARP; P^+^: High expression of HSP10 and low expression of c-PARP.

Elevated expression of HSP10 protein inhibits apoptosis in astrocytoma patients As mentioned above, c-PARP is believed as a featured protein in apoptosis process. Therefore, any factor which can mobilize c-PARP overexpression will basically promote cell apoptosis. On the contrary, it’s understandable that apoptosis has been inhibited when c-PARP protein was driven to lower expression. Spearman's correlation analysis had been performed to investigate the dynamic relationship between elevated expression of HSP10 and apoptosis. Data shown in the Table 2 indicated that elevated HSP10 was significantly negative related to lower expression of c-PARP in astrocytoma (r = -0.224, *P* = 0.023). In another word, aberrant high expression of HSP10 inhibited apoptosis in astrocytoma.

**Table 2:** The pairwise association between expression of HSP10 and c-PARP proteins in the 103 cases of astrocytoma.

	c-PARP	
	Low expression	High expression
**HSP10**		
Low expression	32(31.1%)	19(18.4%)
High expression	43(41.7%)*	9(8.7%)

* Spearman's correlation analysis, R = -0.224, p = 0.023

### Impact of expression of HSP10 and c-PARP proteins on the prognosis of astrocytoma patients

**Fig 3:**
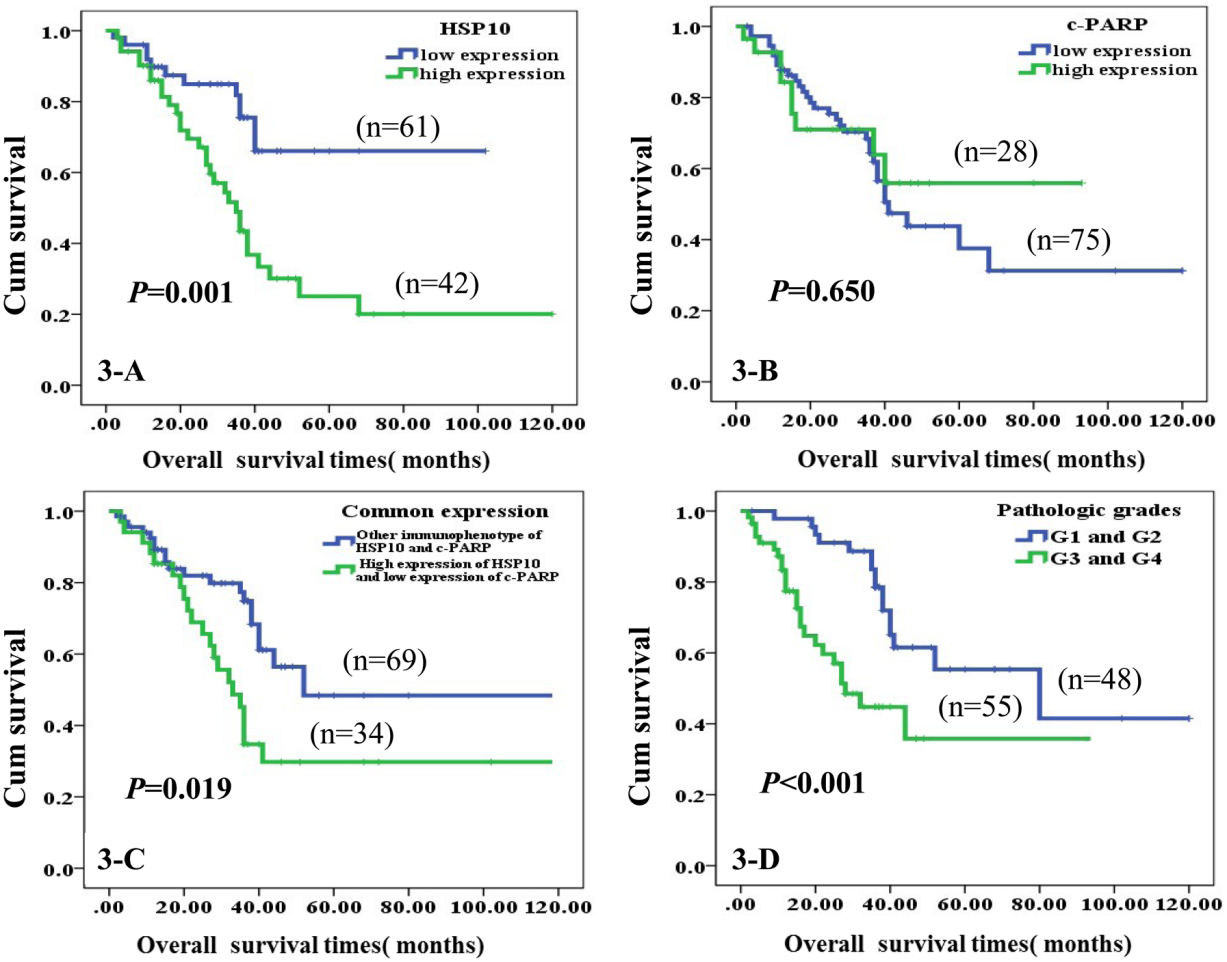
Kaplan-Meier curves according to expression of HSP10 and c-PARP proteins and common expression of two proteins divided into high and low expression. A: High expression of HSP10 was significantly correlated to poor prognosis of astrocytoma patients (P = 0.001, two sided). B: High expression of c-PARP did not significantly relate with survival of astrocytoma patients (*P* = 0.650, two sided). C: Astrocytoma patients with high expression of HSP10 and low expression of c-PARP had significantly short survival times (*P* < 0.019, two sided). D: Astrocytoma patients with high pathologic grades were evidently poor overall survival (*P*< 0.001, two sided).

Kaplan-Meier survival cure with log-rank test was used to compare the univariate survival analysis of astrocytomas patients with expression of HSP10 and c-PARP proteins. The results of Fig 3 illustrated the Kaplan–Meier survival plots for astrocytoma with different expression of HSP10, c-PARP, common expression of both proteins and pathological grades. Irrebuttable, there was a significantly lower overall survival rate for astrocytoma patients with high expression of HSP10 protein compared to those with low expression of HSP10 (*P* = 0.001, Fig 3A), and the same with high expression of HSP10 and low expression of c-PARP in the common expression section (*P* = 0.019, Fig 3C). Also, there was an evidently lower overall survival rate for high pathological grade astrocytoma patients than low pathological grade ones (*P*<0.001, Fig 3D). However, expression of c-PARP had no statistically relationship with overall survival rate in patients with astrocytoma (*P* = 0.650, Fig 3B). The multivariate Cox proportional hazard regression analysis was also carried out to further explore whether the expression of HSP10 and c-PARP proteins were the independent prognostic factors for patients with astrocytoma. In multivariate analysis of the 103 cases, we analyzed expression of HSP10 and c-PARP proteins, WHO grades, tumor size, treatment strategy, age and gender. Our results in Table 3 revealed that overexpression of HSP10 protein was identified as an independent poor prognostic factor for astrocytoma (*P* = 0.032), so did WHO grades (*P* = 0.035). Herein, no impact of c-PARP, tumor size, treatment strategy, age and gender was detected on astrocytoma (*P* > 0.05 for all).

**Table 3:** Summary of multivariate analysis of Cox proportional hazard regression for overall survival in 103 cases of astrocytoma.

Parameter	SE	Wald	Sig.	Exp(B)	95.0% CI for Exp(B)	
					Lower	Upper
**HSP10**	0.372	4.622	0.032*	2.224	1.073	4.608
**c-PARP**	0.389	0.070	0.792	1.108	0.517	2.375
**WHO grades**	0.182	4.433	0.035*	1.468	1.027	2.098
**Tumor size**	0.343	0.566	0.452	0.773	0.394	1.513
**Treatment strategy**	0.135	0.071	0.790	0.965	0.740	1.257
**Age**	0.346	2.988	0.084	1.819	0.923	3.583
**Gender**	0.326	1.652	0.199	0.658	0.347	1.246

**Abbreviations:** CI, confidence interval. **Note:** multivariate analysis of Cox regression,

*: p<0.05

## Discussion

Except for the popular function as “molecular chaperone”, HSP10 has been gradually discovered with multiple roles in cell life activities. Like one coin has two sides, reports about HSP10 in the currently available literature reflect mixed sights into clinic research. Firstly, as it’s known to all, HSP10 is irreplaceable in protein assembling and degradation to keep the stability of cells, with the help of HSP60 [9]. But excessive protein synthesis, which is a prevalent feature of a variety of tumors, would occur after overexpression of a “chaperone”. Then helpfully vigorous anti-autoimmunity of HSP has been carried out. For example, in an allogeneic graft model, recombinant human HSP10 was demonstrated to be capable of prolonging the skin graft survival time [31]. And Hsp10 play a critical role in inhibiting the inflammatory responses to a number of noxae or stressors [32,33]. However, Hsp10 is increasingly reported to be involved, for many reasons, in the pathogenesis and the progression of different human neoplasms and tumors. As we mentioned above, probably during cellular evolution, HSP10 has eventually acquired various functions such as participating in immune system regulation, cell proliferation and differentiation, and, carcinogenesis in particular. Specifically, higher HSP10 levels have been detected in different malignancies such as large bowel cancer [13, 28], hepatocellular carcinoma [14], exocervical cancer [17], serous ovarian cancer [18], mantle cell lymphoma [19] and prostate cancer [34], while lower expression levels of this protein have been discovered in bronchial cancer [35].

In our present study, we explored the association between the expression of HSP10 and c-PARP and clinicopathological characteristics of astrocytoma. We found an overall 40.8% high expression rate for HSP10 in astrocytoma tissues. Moreover, we detected HSP10 in 43 cases of non-tumorous brain tissues and obtained 18.6% of high expression cases. The percentage of high expression of HSP10 was significantly higher in astrocytoma than in the non-tumor control brain tissues (p<0.05), which is in agreement with many other studies. What’s more, high expression of HSP10 was significantly negative related to lower expression of c-PARP in astrocytoma. This means high HSP10 expression can inhibit apoptosis. The most meaningful result in our study was that high expression of HSP10 was definitely an independent poor prognostic factor for astrocytoma regardless of tumor size, treatment strategy, age and gender. Thus, high expression of HSP10 might be a novel biomarker to predict poor prognosis for astrocytoma patients.

The mechanisms and effects of the increased expression of HSP10 in astrocytoma remain a little mysterious. To our knowledge, the most potential molecular mechanism, by which HSP10 induces qualitative changes of brain tissue in astrocytoma, should be the down-regulation of apoptosis pathway to promote cell survival and tumorigenesis. It is, as we figured out, the powerful evidence that high expression of HSP10 was significantly negative related to lower expression of c-PARP, the product of apoptosis process, in astrocytoma. Normally, the majority of HSP10 localizes in the mitochondrial matrix, whereas in tumor cells, overexpressed HSP10 protein aggregates in the cytosol reaching a much higher concentration [36], which is consistent with our IHC results. Accumulated HSP10 protein in cytosol interacts with RAF signaling cascade, inactivates proapoptotic Raf-ERK signaling axis, further inhibit the proapoptotic activity, shifting the balance towards proliferation and cell survival [37]. While according to the studies of Shan and colleagues, Bcl-2/Bax pathway is also involved in the anti-apoptosis process. The overexpression of HSP10 increases the abundance of antiapoptotic Bcl-2 and Bcl-xl (Bcl-x protein long isoform), prominently causing decreased protein expression of the proapoptotic Bax (Bcl-2-associated X protein). The overexpression of HSP10 then stabilizes mitochondrial cross-membrane potential, inhibits caspase-3, and suppresses poly(5′-diphosphate-ribose) polymerase [38]. However, whether HSP10 influences apoptosis via Raf signaling cascade or Bcl-2/Bax pathway or others in astrocytoma is continually needed to be further verified. Certainly, roles of HSP10 in tumorigenesis will never limit to inhibit apoptosis. A review has confirmed that Hsp10 is an active player of cell signaling network, influencing cell cycle, nucleocytoplasmic transport, and metabolism, which would be important cancer etiology [15]. Another report proves that HSP10 overexpression increases the number of functioning receptors and amplified activation of insulin-like growth factor-1R signaling, which is implicated in several cancers [39]. In addition, cancer progression may intensify if immune function is suppressed. HSP10 appears to suppress T-cell expression of CD3-zeta, a key component of T-cell activation, and then stops T-cell activation, allowing the tumor to escape immune surveillance [40]. Actually, the exact effect of HSP10 in astrocytoma may vary from other tumors. Besides, immunohistochemistry staining as the only method to detect proteins in tissues has its limitations. Thus, we are looking forward to find out the accurate pathway through which HSP10 inhibits apoptosis in the future, and, we can also test the effect of monoclonal antibodies against HSP10 on astrocytoma.

Meanwhile, back to astrocytoma itself, it’s a special kind of tumor characterized with star-like tumor cells. Tumor cells in astrocytoma extend ultra-long membrane protrusions, which is consisted with well-developed tumor microtubes system. Membrane tube formation is a novel means of tumor dissemination. These distinct tumor microtubes serve as routes for brain invasion, proliferation, and to interconnect over long distances [41]. Abundant tumor intercellular membrane tubes are actin-rich and interestingly, mitochondria is observed travelling quickly in these tubes under scanning electron microscopy [41]. We suppose that the elevated expression of HSP10 enhances the assembly of tumor microtubes system remarkably, and then HSP10 would be a promising target to restrain astrocytoma progression. This would be of brilliant worth for investigation in our future studies.

In summary, we firstly report that there was high expression of HSP10 protein in astrocytoma. High HSP10 expression can inhibit apoptosis and it was closely associated with shorter overall survival in astrocytoma patients. So, high expression of HSP10 protein might serve as a novel independent biomarker for predicting a poor prognosis in patients with astrocytoma.
